# A Luneburg Lens Antenna for High-Speed Railway Communication

**DOI:** 10.3390/mi17070820

**Published:** 2026-07-07

**Authors:** Qiao-Na Qiu, Dong Yang, Jun Wang

**Affiliations:** 1 School of Intelligent Manufacturing, Jiaxing Vocational and Technical College, Jiaxing 314036, China; 2 Key Laboratory of RF Circuits and System of Ministry of Education, Hangzhou Dianzi University, Hangzhou 310018, China; yangdong9602@163.com; 3 School of Information and Control Engineering, China University of Mining and Technology, Xuzhou 221116, China

**Keywords:** 1800/FA band antenna, luneburg lens, base station antenna

## Abstract

To address the problems in high-speed railway communication, such as large signal penetration loss through carriages, difficulty in long-distance strip coverage, and limited coverage range of traditional base station antennas, this paper designs a cylindrical Luneburg lens antenna operating at the 1800/FA frequency bands. A dual-polarized feed antenna with a dipole structure is designed, loaded with X-shaped metal strips for out-of-band suppression, and integrated with a four-layer dielectric stratified cylindrical Luneburg lens, which uses its graded permittivity distribution to achieve beam focusing, enhance gain, narrow the horizontal beamwidth, and maintain a wide vertical beamwidth. Simulation results show that the lens can stably improve the gain by about 5 dBi; measured results indicate that the antenna has port isolation higher than 35 dB, good impedance matching, and measured gain of 12.4–13.3 dBi within the 1.7–2.1 GHz band, which is highly consistent with the simulation. This antenna can effectively adapt to the long-distance strip coverage scenario along high-speed railways, reduce the base station deployment density, and provide an engineering solution for the optimization of high-speed railway communication coverage.

## 1. Introduction

China owns the longest high-speed railway mileage in the world, and high-speed railways have become the core strength of China’s comprehensive transportation system [1]. High-speed railways feature high operating speed, large passenger flow, and complex terrain and deployment scenarios along the lines. Providing continuous, stable and high-quality mobile communication coverage under the conditions of high-speed train operation and prominent shielding effect of enclosed carriages has become a key problem urgently to be solved in the field of modern wireless communication [2]. High-speed railway carriages adopt fully enclosed structures made of stainless steel or aluminum alloy, which significantly increases the penetration loss of wireless signals. Meanwhile, the long-distance linear layout of high-speed railway lines requires the communication system to achieve long-range continuous coverage [3].

To realize seamless and high-quality communication coverage along high-speed railways, the adopted base station antennas need to effectively overcome the severe signal penetration loss caused by metal carriages and possess beam characteristics suitable for strip coverage. A reasonable beamwidth design is required to meet the demand of long-distance continuous coverage along the lines [4]. Traditional panel base station antennas generally adopt a large-scale vertical array arrangement to improve radiation gain, which results in a narrow vertical half-power beamwidth and limits the effective coverage distance and radiation range of a single base station. The dense deployment of base stations is essential to ensure full continuous coverage along high-speed railways, which not only increases project construction and operation maintenance costs but also easily causes signal overlap and frequent handover between adjacent stations [5]. In addition, traditional panel antennas have narrow vertical beamwidth, which tends to produce vertical and horizontal coverage holes in high-speed railway coverage and cannot meet the continuous coverage requirements under high-speed mobile scenarios [6]. In addition, conventional panel antennas suffer severe signal fluctuations under an obvious Doppler shift brought by high-speed train operation.

In recent years, lens antennas have gradually become a research hotspot for high-speed railway communication coverage due to their unique performance advantages [7,8,9,10]. As shown in Reference [11], compared with traditional panel antennas, the vertical beamwidth of lens antennas can reach 23° to 30°, more than three times that of traditional antennas. Lens antennas can effectively extend the antenna coverage distance, improve the spectral efficiency and communication quality of the system, and reduce the number of base stations. Among them, Luneburg lens antennas possess inherent advantages such as a concise structure, high radiation gain and wide vertical beamwidth, showing good adaptability to long and narrow strip coverage scenarios [12,13,14,15,16], and they are more suitable for the practical application of mobile communication along high-speed railways than traditional panel base station antennas [17].

Combining the beam regulation advantages of cylindrical Luneburg lens and the design characteristics of a compact dual-band base station antenna array [18], this paper develops an 1800/FA dual-band Luneburg lens antenna suitable for base station deployment along high-speed railways. Making full use of the inherent beam-focusing characteristic of the cylindrical Luneburg lens, the proposed antenna effectively narrows the horizontal half-power beamwidth, improves the overall radiation gain, suppresses the co-channel adjacent interference, and reduces the handover interference level between base stations, alleviating the difference in propagation loss induced by complex terrain. At the same time, the antenna maintains a wide vertical beam coverage range, which can effectively adapt to the long-distance strip coverage scenario along high-speed railways [19]. It reduces the deployment density of base stations while ensuring communication quality, saves network construction and operation costs, and provides a feasible solution for the optimal networking and coverage improvement of communication networks along high-speed railways [20].

## 2. Design of the 1800/FA Band Feed Antenna

To meet the coverage requirements of mobile communication systems along high-speed railways, the feed antenna must support both the 1800 MHz and FA bands. Accordingly, this paper presents an antenna design using a loop dipole as the main radiator, enabling effective coverage of the wide operating bandwidth across the 1800 MHz and FA bands.

**Figure 1:**
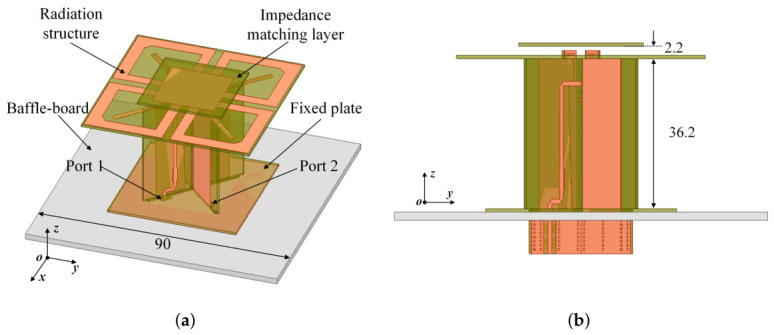
Feed antenna model for 1800/FA band (Unit: mm): (**a**) 3D view of the feed antenna, (**b**) side view of the feed antenna.

The model of the proposed feed antenna operating in the 1800/FA band is illustrated in Figure 1. The antenna comprises four core components: the radiation structure, impedance matching layer, feeding balun and fixing plate. It features a compact configuration and facilitates fabrication and assembly.

The radiation structure of the antenna adopts a pair of orthogonally arranged loop cross dipoles, which are placed along the $\pm 45^{\circ }$ direction to form a dual-polarized radiating element. Dual linear polarization operation can be realized by exciting two independent ports. To broaden the operating bandwidth of the antenna, the loop oscillator is designed with an extended current path, enabling it to cover the wideband requirements of both the 1800 MHz and FA bands.

The proposed antenna adopts an FR4 dielectric substrate as the PCB material, with a relative permittivity of $\varepsilon_{r}=4.3$, a loss tangent of tanδ=0.01, and a substrate thickness of 0.76 mm. Such a selection balances the fabrication cost and electrical performance. An impedance matching layer is arranged above the radiation structure. By adjusting its dimensions and spacing, the input impedance characteristics of the antenna are optimized to achieve good matching with the feeding system. The feeding part of the antenna employs a balanced feeding balun structure, which converts the unbalanced feeding of the coaxial line into the balanced feeding required by the dipole. Meanwhile, impedance transformation is realized, the spurious radiation of the feed line is suppressed, and high isolation between the dual-polarized ports is ensured.

**Figure 2:**
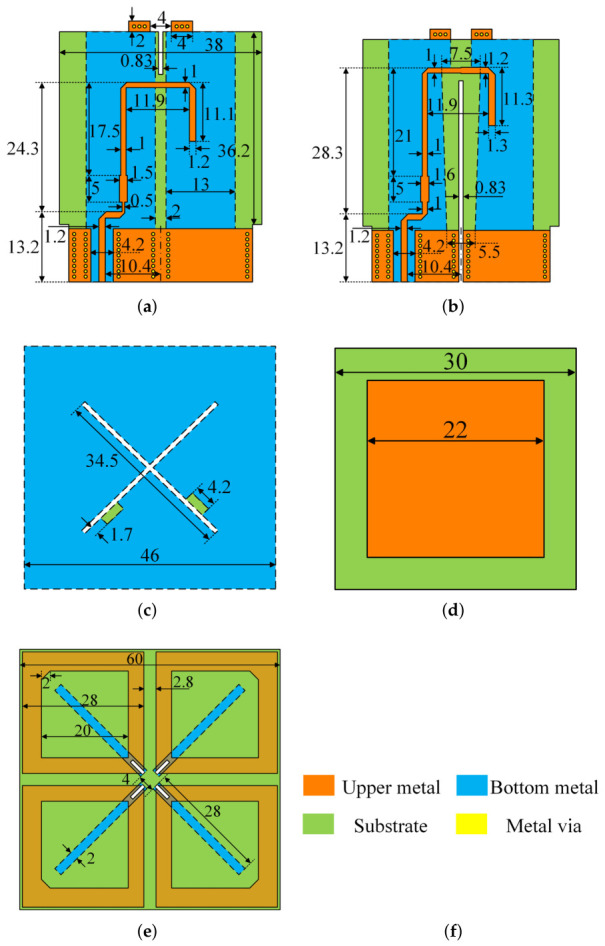
Specific structure and dimensions of the 1800/FA band feed antenna (Unit: mm): (**a**) Balun fed by Port 1. (**b**) Balun fed by Port 2. (**c**) Fixing plate. (**d**) Impedance matching layer. (**e**) Radiation structure. (**f**) Legend of the structural schematic.

A square metal reflector with a side length of 90 mm is arranged at the bottom of the antenna, and its vertical distance from the radiation structure is 36.2 mm. It is utilized to reflect the backward radiated energy, enhance the forward gain of the antenna, and form a directional radiation pattern. The fixing plate is used to fasten the feeding balun and radiating elements, maintaining stable relative positions of all components and avoiding degradation of electrical performance caused by structural deformation. After full-wave simulation and parametric optimization of the antenna, the detailed dimensions of each component are finally determined, and the specific parameters are presented in Figure 2.

**Figure 3:**
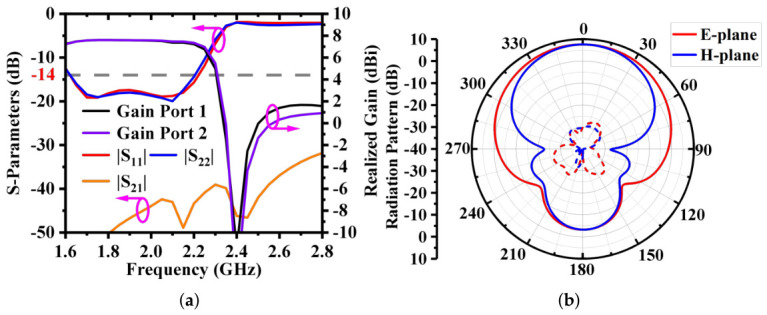
Simulation results of the feed antenna: (**a**) Simulated S-parameters and gain of the 1800/FA band feed antenna. (**b**) Simulated radiation patterns of the 1800/FA band feed antenna at 1.9 GHz.

In this paper, simulation analysis is carried out on the key parameters of the aforementioned 1800/FA band feed antenna, including the S-parameters, gain, and radiation patterns, and the simulation results are presented in Figure 3.

As can be observed from the S-parameter curves in Figure 3a, the two ports of the antenna exhibit excellent performance consistency. Within the frequency range of 1.6–2.2 GHz, the reflection coefficients of both ports are lower than −14 dB, corresponding to a voltage standing wave ratio (VSWR) of less than 1.5. Meanwhile, the isolation between the two ports exceeds 40 dB, demonstrating superior polarization isolation performance. When each port is excited independently, the antenna gain remains stably around 7.5 dBi across the entire operating band of 1.6–2.2 GHz with small gain fluctuation and favorable broadband stability. Benefiting from the notch design adopted in the radiation structure, the antenna gain is suppressed below 2 dBi in the frequency range higher than the operating band, which achieves excellent out-of-band suppression and effectively reduces interference to other communication frequency bands. Figure 3b plots the radiation pattern of the antenna when Port 1 is excited alone. It can be seen that the antenna achieves stable directional radiation characteristics with a low cross-polarization level, and its radiation performance meets the design expectations.

**Figure 4:**
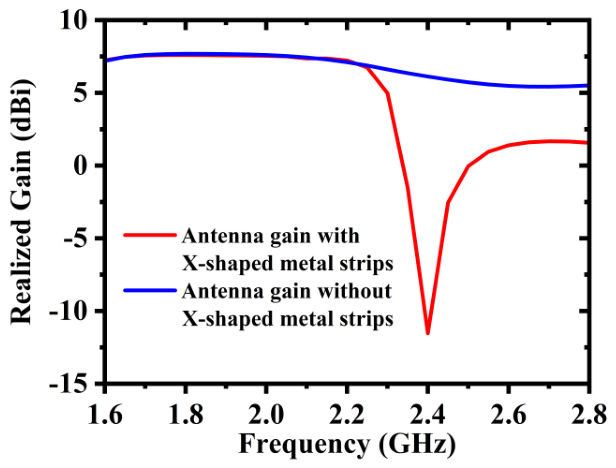
Gain before and after loading X-shaped metal strips.

Meanwhile, to improve the out-of-band suppression capability of the antenna, X-shaped metal strips are loaded on the bottom layer of the dielectric substrate. Dense concentrated induced currents can be excited on the surface of the X-shaped metal strips, which equivalently form anti-phase radiating elements to counteract the electromagnetic waves radiated outward by the main radiating vibrators. As a result, deep radiation nulls are formed at the target out-of-band frequency point to achieve favorable out-of-band suppression performance. Benefiting from this notch characteristic, extra radiation nulls are introduced in undesired frequency bands, lowering the out-of-band gain and mitigating interference to other communication frequency bands. The detailed comparison is shown in Figure 4.

Overall, the comprehensive performance of the proposed feed antenna fully satisfies the design specifications of the lens antenna, which can provide reliable support for subsequent system integration.

## 3. Design of Luneburg Lens Antenna

### 3.1. Cylindrical Luneburg Lens

**Figure 5:**
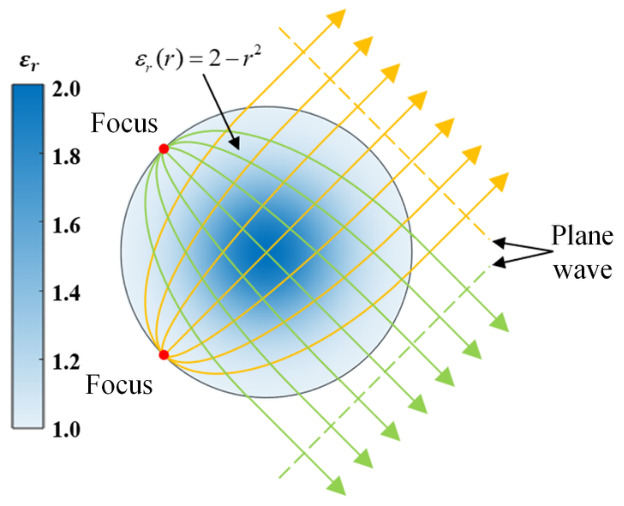
Schematic diagram of the working principle of Luneburg lens.

A standalone feed suffers from insufficient gain and a wide beamwidth, making it unsuitable for high-speed railway communication scenarios. The lens antenna can be combined with a feed antenna to effectively solve the vertical coverage hole problem in high-speed railway coverage, and it has significant advantages in improving the single-station coverage range, reducing the number of station sites, and lowering the construction, operation and maintenance costs. The Luneburg lens is a typical lens antenna, and its working principle is illustrated in Figure 5. Ideally, the radial permittivity of an ideal Luneburg lens follows a continuous parabolic distribution. However, it is impossible to fabricate continuously gradient dielectric materials in practical engineering. Accordingly, a discrete layered approximation scheme is adopted, where the theoretical gradient curve is approximated segmentally by multiple layers of uniform dielectric substrates. The focal point of the lens is located on its surface, and the internal dielectric constant presents a gradient distribution with the radius, which can accurately regulate the phase of electromagnetic waves and effectively convert the spherical waves emitted from the focal point into plane waves [21].

The dielectric constant distribution inside the lens gradually varies from 2 at the center to 1 at the surface, and its specific distribution is given by

(1) $$\varepsilon_{r}\left(r\right)=2-r^{2}$$

where *r* is the normalized radius at a certain position in the lens, and $\varepsilon_{r}\left(r\right)$ is the relative permittivity at that position.

The cylindrical Luneburg lens antenna is derived from the spherical Luneburg lens antenna. Analogous to the three-dimensional problem of the spherical Luneburg lens, the cylindrical Luneburg lens can be simplified into a two-dimensional problem and is thus also referred to as a two-dimensional Luneburg lens antenna [22]. Theoretically, any point on the cylindrical surface can serve as the focal point of the lens. Compared with the spherical Luneburg lens antenna, the cylindrical Luneburg lens antenna exhibits a radiation pattern with a narrow beam in the azimuth plane and a wide beam in the elevation plane, offering significant advantages in wide-angle scanning performance over other multi-beam antennas [23].

**Figure 6:**
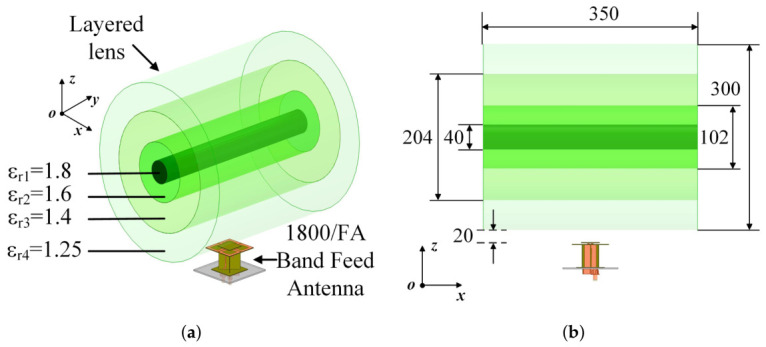
1800/FA band Luneburg lens antenna model and dimensions (mm): (**a**) 3D model of the lens antenna. (**b**) Side view of lens antenna.

Therefore, in this paper, a cylindrical multi-layer dielectric Luneburg lens composed of four materials with different dielectric constants is adopted, as shown in Figure 6a. The dielectric layers of the proposed four-layer cylindrical Luneburg lens are fabricated from physically foamed polyethylene. Relying on precise foaming ratio control technology, four stable relative permittivity values of 1.25, 1.4, 1.6 and 1.8 are realized. All four layers take polyethylene as the substrate matrix. By adjusting the injection pressure of supercritical fluid and extrusion rate, the proportion of internal air micropores is modulated; so, the gradient permittivity design can be realized without doping high-permittivity fillers. From the outside to the inside, the lens consists of dielectric layers with relative permittivities of 1.25, 1.4, 1.6, and 1.8, and the corresponding radii of each layer are 48 mm, 51 mm, 31 mm, and 20 mm, respectively.

### 3.2. Luneburg Lens Antenna

The configuration of the proposed Luneburg lens antenna is illustrated in Figure 6, which mainly consists of two parts: a layered lens and a feed antenna. The feed antenna employs the 1800/FA band feed element, while the layered lens is the cylindrical Luneburg lens presented in Figure 6a. Loading this lens can effectively narrow the beamwidth in the azimuth plane (yoz-plane, the same below) and enhance the antenna gain. With identical transmit power, the antenna can generate stronger field strength for long-distance coverage, which compensates for signal attenuation caused by the shielding effect of metal train carriages.The antenna is optimized in simulation software, and the final dimensions are determined as shown in Figure 6b.

**Figure 7:**
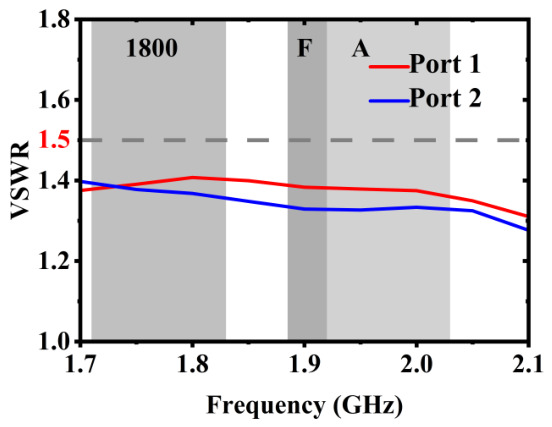
VSWR of the two ports of the Luneburg lens antenna.

The voltage standing wave ratio (VSWR) characteristics of the two ports of the 1800/FA band luneburg lens antenna within the 1.7–2.1 GHz frequency range are presented in Figure 7. It can be observed that the VSWR values of both ports remain stable between 1.25 and 1.41 across the entire band, satisfying the excellent impedance matching criterion of VSWR ≤1.5. This indicates that the antenna achieves efficient energy transmission with extremely low reflection loss throughout the operating bandwidth. Meanwhile, the VSWR curves of the two ports exhibit highly consistent trends with only minor deviations, verifying good consistency and symmetry between the antenna ports.

**Figure 8:**
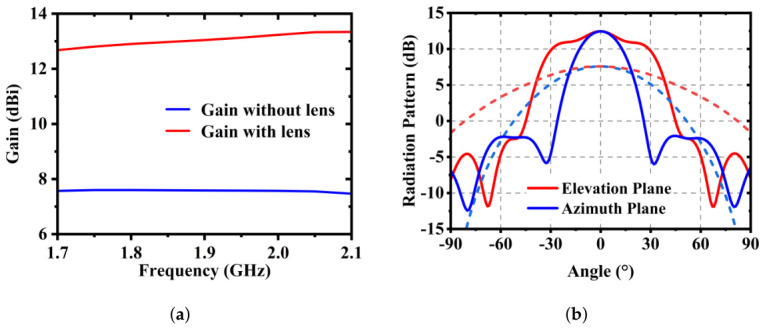
Simulation results of 1800/FA band Luneburg lens antenna: (**a**) Gain comparison. (**b**) Radiation pattern comparison.

The gain characteristics of the antenna with and without the cylindrical Luneburg lens in the frequency range of 1.7–2.1 GHz are compared in Figure 8a. Without the lens, the gain of the feed antenna is stable at approximately 7.5 dBi; with the lens loaded, the antenna gain increases to 12.7–13.3 dBi, achieving a stable gain improvement of about 5 dBi, which verifies the excellent beam focusing and energy converging capability of the lens. Figure 8b compares the two-dimensional radiation patterns of the antenna with and without the cylindrical Luneburg lens, where the solid lines represent the patterns with the lens, and the dashed lines correspond to the feed antenna without the lens. It can be seen that, after loading the lens, the peak gain of the main lobe in both the azimuth and elevation planes is significantly improved, with an increase of approximately 4–5 dB compared to the case without the lens, further confirming the lens’s superior beam focusing performance. Meanwhile, the beamwidth in the azimuth plane is highly narrowed, with the half-power beamwidth significantly smaller than that without the lens, while the beamwidth in the elevation plane is also compressed to a certain extent. The overall radiation pattern forms a directional shape with “narrow azimuth and wide elevation”, which is suitable for the typical coverage requirements of base station antennas. In addition, the sidelobe levels in both planes are effectively suppressed after loading the lens, remaining below −10 dB overall, with good symmetry and no obvious distortion. This indicates that the introduction of the lens not only improves the main lobe gain but also effectively enhances the radiation directivity and anti-interference capability of the antenna.

## 4. Results and Discussion

**Figure 9:**
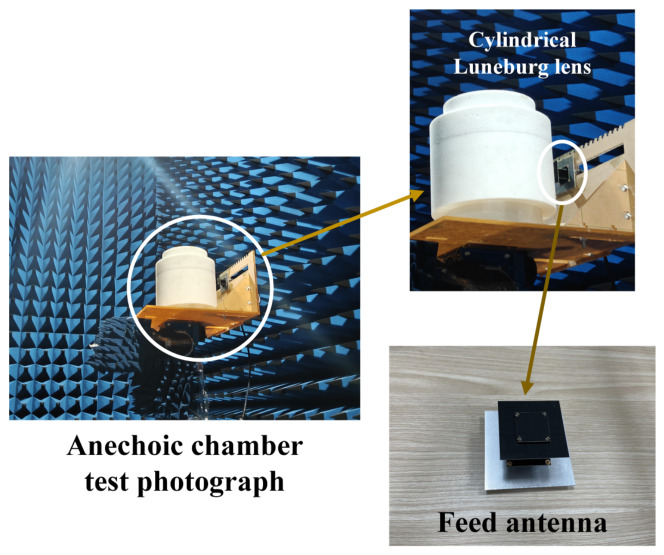
Structural configuration and physical prototype photograph of cylindrical Luneburg lens antenna.

To verify the feasibility and effectiveness of the proposed design concept, a prototype of the antenna was designed, fabricated, and measured in this paper. Figure 9 presents the anechoic chamber test photograph of the Luneburg lens antenna array prototype, which mainly consists of two parts: a cylindrical Luneburg lens and a feed source. Among them, the cylindrical Luneburg lens adopts the four-layer layered structure with different dielectric constants described in the previous section; the feed source is selected as a base station antenna operating in the 1800/FA band, and the surface of the feed PCB is coated with a dielectric anti-corrosion protective layer to enhance environmental adaptability. A structure form combining a single feed source with the cylindrical Luneburg lens is adopted in this paper, and its specific structure is shown in Figure 9.

### 4.1. 1800/FA Band Feed Antenna

**Figure 10:**
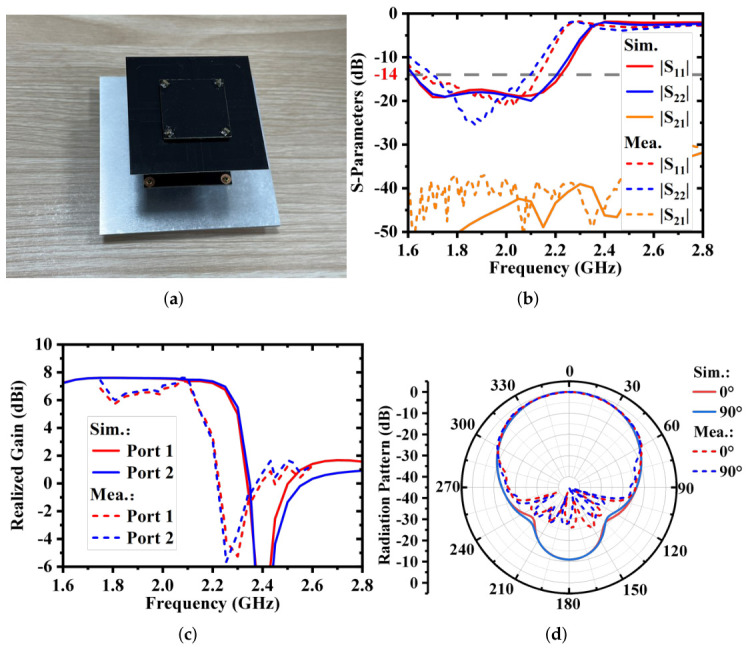
Simulated and measured results of 1800/FA band feed antenna: (**a**) Prototype photo. (**b**) S-parameters. (**c**) Gain. (**d**) Radiation pattern at 1.9 GHz.

The 1800/FA band feed antenna is tested individually in this work. Figure 10a shows the photograph of the fabricated 1800/FA-band feed antenna. The antenna is manufactured using PCB technology and assembled into an integrated structure. Considering the anti-corrosion requirements in practical engineering applications, a protective layer is added onto the antenna surface.

Figure 10b–d present the comparison between the simulated and measured results of the 1800/FA band feed antenna. The fabricated feed PCB is coated with a dielectric anti-corrosion protective layer absent in the ideal simulation model; the high permittivity coating increases the equivalent electrical length, shifting resonance downward and introducing extra dielectric loss to reduce measured gain overall. It can be observed that the measured −14 dB operating bandwidth (corresponding to a voltage standing wave ratio of 1.5) is 1.69–2.10 GHz, which is slightly narrower than the simulated result but still covers the designed operating frequency band. Within the operating band, the measured port isolation is higher than 35 dB, demonstrating excellent isolation performance. The measured gain ranges from 5.8 to 7.6 dBi, which is 0–1.8 dB lower than the simulated result. In addition, the measured notch frequency exhibits an obvious shift compared with the simulation, but it still lies outside the operating band. The measured radiation patterns show good performance, with the beamwidth slightly wider than the simulated results.

The discrepancies between simulated and measured results can be attributed to the combined effect of fabrication errors and the anti-corrosion layer on the antenna surface. The material of the anti-corrosion layer has a relative permittivity of $\varepsilon_{r}=3.9$ and a loss tangent of tanδ=0.03, which introduces considerable loss.

### 4.2. 1800/FA Band Luneburg Lens Antenna

**Figure 11:**
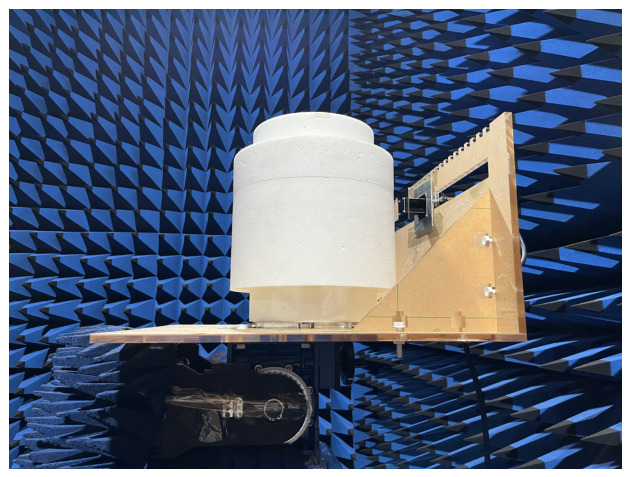
Prototype photograph of cylindrical Luneburg lens antenna.

The fabricated cylindrical Luneburg lens antenna operating at 1800/FA bands is experimentally characterized. The photograph of the measurement setup is shown in Figure 11.

**Figure 12:**
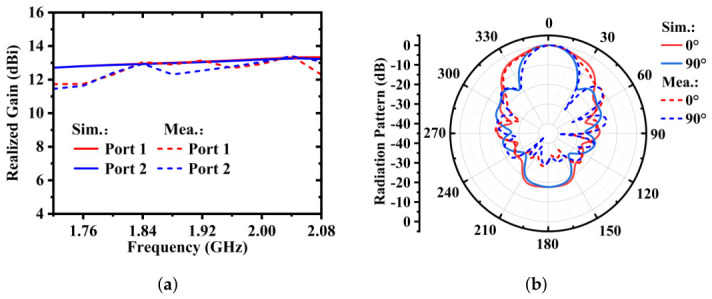

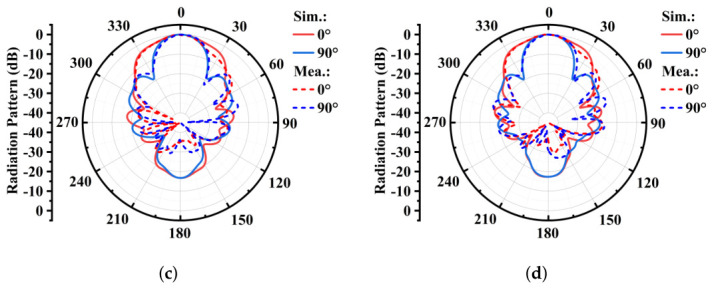
Simulated and measured results of 1800/FA band Luneburg lens antenna: (**a**) Gain. (**b**) Radiation pattern at 1.8 GHz. (**c**) Radiation pattern at 1.9 GHz. (**d**) Radiation pattern at 2.0 GHz.

Figure 12a compares the simulated and measured realized gains of the two ports of the Luneburg lens antenna across different frequencies. Within the frequency range of 1.72–1.84 GHz, the measured gain of both ports is slightly lower than the simulated result, while good agreement between the measured and simulated gains is observed in the band of 1.84–2.05 GHz. It is inferred that the degradation of gain at the low-frequency band is mainly attributed to the deviation between the actual dielectric constant of the lens material and the preset value in simulation, which deteriorates the beam focusing performance and deviates from the theoretical expectation. Correspondingly, Figure 12b–d illustrate the two-dimensional radiation patterns at 1.8 GHz, 1.9 GHz and 2.0 GHz. After loading the cylindrical Luneburg lens, the realized gain of the antenna is significantly improved. Specifically, the gain increases from 7.60 dBi to 12.4 dBi at 1.8 GHz, from 7.58 dBi to 12.95 dBi at 1.9 GHz, and from 7.57 dBi to 13.3 dBi at 2.0 GHz, which verifies the excellent beam focusing capability of the lens. The radiation patterns at each frequency point feature a well-shaped main lobe and low sidelobe level, demonstrating satisfactory directional radiation performance and radiation efficiency of the antenna within the entire operating band. Simulated (solid line) and measured (dashed line) results achieve high consistency in terms of main lobe direction, peak gain and half-power beam width. Only minor discrepancies exist in the sidelobe region, which are mainly caused by fabrication tolerance, assembly error and measurement environment.

## 5. Conclusions

This paper combines the advantages of Luneburg lenses and compact base station antenna. Targeting the engineering requirements of base stations along high-speed railways, a 1800/FA band lens antenna is developed through scheme design, structural optimization, and experimental verification. First, an 1800/FA band feed antenna is designed. Then, a layered cylindrical Luneburg lens is integrated to achieve beam focusing and gain enhancement, forming the final lens antenna. The measured results show that the antenna exhibits excellent radiation performance across 1.7–2.1 GHz, with port isolation higher than 35 dB and good impedance matching. The measured gain ranges from 12.4 to 13.3 dBi, which is in good agreement with simulations, verifying the feasibility and reliability of the proposed design. Featuring high gain, narrow azimuth beamwidth, wide elevation coverage, compact structure, and easy integration, the antenna holds promising engineering application value and broad prospects for communication and base station coverage along high-speed railways.

## Data Availability

The original contributions presented in this study are included in the article. Further inquiries can be directed to the corresponding author.
